# Precise co-registration of mass spectrometry imaging, histology, and laser microdissection-based omics

**DOI:** 10.1007/s00216-019-01983-z

**Published:** 2019-07-01

**Authors:** Frédéric Dewez, Marta Martin-Lorenzo, Michael Herfs, Dominique Baiwir, Gabriel Mazzucchelli, Edwin De Pauw, Ron M.A. Heeren, Benjamin Balluff

**Affiliations:** 1 Maastricht Multimodal Molecular Imaging Institute (M4I), Maastricht University, Universiteitssingel 50, P.O. Box 616, 6200 MD Maastricht, The Netherlands; 2 Mass Spectrometry Laboratory (L.S.M), University of Liège, 4000 Liège, Belgium; 3 Laboratory of Experimental Pathology, GIGA-Cancer, University of Liège, Avenue de l’Hôpital 11, 4000 Liège, Belgium

**Keywords:** Mass spectrometry imaging, Laser microdissection, Microproteomics, Co-registration, Intratumor heterogeneity

## Abstract

Mass spectrometry imaging (MSI) is an analytical technique for the unlabeled and multiplex imaging of molecules in biological tissue sections. It therefore enables the spatial and molecular annotations of tissues complementary to histology. It has already been shown that MSI can guide subsequent material isolation technologies such as laser microdissection (LMD) to enable a more in-depth molecular characterization of MSI-highlighted tissue regions. However, with MSI now reaching spatial resolutions at the single-cell scale, there is a need for a precise co-registration between MSI and the LMD. As proof-of-principle, MSI of lipids was performed on a breast cancer tissue followed by a segmentation of the data to detect molecularly distinct segments within its tumor areas. After image processing of the segmentation results, the coordinates of the MSI-detected segments were passed to the LMD system by three co-registration steps. The errors of each co-registration step were quantified and the total error was found to be less than 13 μm. With this link established, MSI data can now accurately guide LMD to excise MSI-defined regions of interest for subsequent extract-based analyses. In our example, the excised tissue material was then subjected to ultrasensitive microproteomics in order to determine predominant molecular mechanisms in each of the MSI-highlighted intratumor segments. This work shows how the strengths of MSI, histology, and extract-based omics can be combined to enable a more comprehensive molecular characterization of in situ biological processes.

**Electronic supplementary material:**

The online version of this article (10.1007/s00216-019-01983-z) contains supplementary material, which is available to authorized users.

## Introduction

Mass spectrometry imaging (MSI) is a powerful tool for the non-labeled and parallel imaging of hundreds to thousands of molecules in a single biological tissue section. It allows obtaining cell type–specific molecular patterns and conversely the annotation of tissues based on their molecular profiles [1]. The latter capability has, for instance, been used for the chemical and spatial descriptions of tumors to reveal molecularly distinct tumor cell populations [2].

Image co-registration has already performed in MSI. This has enabled researchers increasing the MSI resolution mathematically to fuse 3D MSI data to the MRI space, to align multiple MSI datasets from a single sample, or to guide MSI experiments based on the tissue’s morphology [3–6]. Likewise, few studies have already used MSI to guide laser microdissection (LMD) systems to isolate regions of interest (ROIs) from the target tissue, but no information on the accuracy of spatial co-registration has been evaluated or reported [7, 8]. Since MSI now routinely reaches a spatial resolution of 10 μm on commercial systems, the co-registration accuracy becomes crucial in order to retain the detailed spatial information provided by MSI in the LMD system.

Here we present an accurate co-registration (considering the currently achievable spatial resolution) of MSI to LMD on the same tissue section and after hematoxylin and eosin staining. We will show how this system can be used to comprehensively and accurately characterize tumor subpopulations defined by MSI, using subsequent microproteomics.

## Experimental section

### Tissue material

Residual (fresh) breast cancer tissue was collected by the Tissue Biobank of the University of Liege, directly frozen in liquid nitrogen, and then stored at − 80 °C. The standardized protocol was approved by the Ethics Committee of the University Hospital Center of Liege. Informed consent was obtained from the participant included in this study. A cryosection of 12-μm thickness was thaw-mounted on a polyethylene naphthalate (PEN) membrane slide (Leica Microsystems, Wetzlar, Germany) and stored at − 80 °C until analysis.

### Mass spectrometry imaging

All solvents, if not stated otherwise, were purchased from Biosolve (Dieuze, France). First, the membrane slide was desiccated for 30 min at room temperature. Several fiducial markers were applied next to the tissue using water-based Tipp-Ex (BIC, Paris, France) for later co-registration purposes. Five milligrams per milliliter α-cyano-4-hydroxycinnamic acid (Sigma Aldrich, St. Louis, MO, USA) in 70% acetonitrile and 0.2% trifluoroacetic acid was sprayed in eight layers using an HTX-TM sprayer (Chapel Hill, NC, USA) onto the tissue section with a constant flow rate of 0.1 ml/min and at a speed of 1300 mm/min. The breast cancer section was measured with a MALDI HDMS SYNAPT G2-Si (Waters, Manchester, UK) which is compatible with non-conductive PEN membrane slides. The experiment was performed in positive mode and at 70-μm spatial resolution within a mass range of *m/z* 350–1600 in which mostly lipids are detected. Red phosphorus was used for external calibration.

### Staining and optical images

Directly after the MSI experiment, a digital high-resolution image of the slide with matrix was obtained with a microscopic slide scanner (Mirax Desk, Zeiss, Jena, Germany), subsequently referred to as the optical image.

Then, the matrix was removed with 70% ethanol and stained for hematoxylin and eosin (H&E) used as standard protocol (Milli-Q water 3 min, hematoxylin 90 s, tap water 3 min, eosin 30 s, tap water 3 min, 100% ethanol 1 min, xylene 30 s). The H&E-stained tissue section was not covered with a cover slip but immediately scanned with the same microscopic slide scanner and stored at − 80 °C until LMD. This resulted in a digital optical image, subsequently referred to as the H&E image.

Both digital high-resolution images were downscaled 1:4 for better handling using the scan viewer software (Pannoramic Viewer, 3DHISTECH Ltd., Budapest, Hungary) which resulted in a pixel size of 2.076 μm for *x* and 2.084 μm for *y* (12,235 × 12,189 dpi).

### Co-registration, data analysis, and image processing

**Fig. 1:**
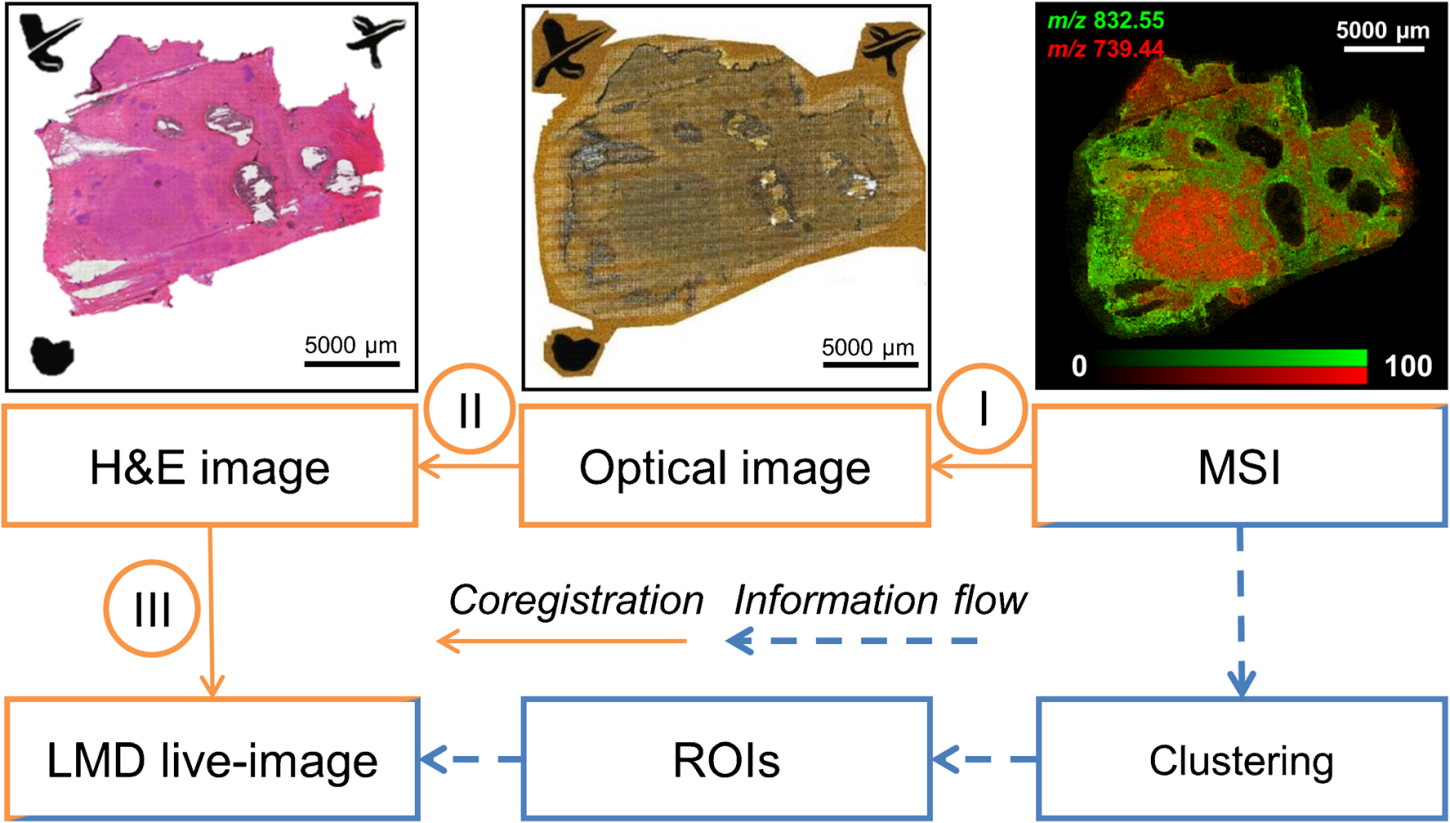
Co-registration steps from mass spectrometry imaging (MSI) to laser microdissection (LMD). Several co-registration steps are needed in order to transfer spatial information from MSI to the LMD system (orange arrows). First, the MSI data was co-registered to the high-resolution optical image of the tissue section via the visible laser shots in the matrix (ESM, Fig. S1) directly after the MSI experiment (I). The optical image was then further matched to its high-resolution H&E image via fiducial markers (II). Finally, coordinates in the H&E image were recalculated using fiducial markers that are both visible in the H&E image and in the LMD live image (III). The established link between MSI, histology, and LMD allows transferring region-of-interest information from MSI, for instance spatial segments identified by a multivariate clustering of the spectra, to the LMD system (blue-dashed arrows)

These images were imported together with the MSI data into MATLAB R2017b (MathWorks, Natick, MA, USA) for co-registration of the images (MSI, optical and H&E; Fig. 1), data analysis, and image processing with the Image Processing toolbox (v.10.1). All image co-registrations were performed using affine geometric transformation (command *fitgeotrans*). Every spectrum of the MSI data was normalized to its total ion current. Tumor-associated spectra were clustered using non-negative matrix factorization (NNMF) where each pixel is assigned to the component with the highest score. After image processing of the segmentation results (see Electronic Supplementary Material (ESM), Figs. S3 and S4), the coordinates of the regions belonging to the segments were recalculated with respect to the coordinates of the fiducial markers in the optical image and written into an XML file for compatibility with subsequent LMD system.

### Laser microdissection

Laser microdissection was performed using a Leica LMD 7000 (Leica Microsystems, Wetzlar, Germany). This system supports the import of external coordinate information of areas to be cut out in form of an XML file. Areas were dissected from the H&E-stained tissue sections with the following settings: wavelength 349 nm, power 20, aperture 45, speed 15, specimen balance 0, head current 100%, and pulse frequency 501 Hz. The microdissected regions were collected in 0.5-ml centrifuge tubes and stored at − 80 °C until further analysis.

### Microproteomics analysis

For every MSI segment, a total of 0.3-mm^2^ dissected material was prepared and analyzed by an optimized LC-MS/MS protocol for bottom-up proteomics of very small samples (~ 2000 cells) using an ultraperformance liquid chromatography (UPLC) 2D nanoACQUITY (Waters, Corp., Milford, USA). This was done as described previously [9], but without paraffin removal and antigen retrieval due to the use of fresh-frozen tissue sections.

Protein identification and label-free quantification (LFQ) were performed using MaxQuant v.1.5.6.5 with the following settings: UniProt reviewed human database, trypsin digestion with maximum two missed cleavage sites, methionine oxidation as variable modification and carbamidomethyl cysteine as fixed modification, a minimal peptide length of seven amino acids, at least two peptides per protein (of which at least one is unique), and a maximum false discovery rate of 1%. The label-free intensities were normalized using the MaxLFQ algorithm [10].

Data analysis was performed with Perseus v.1.6.2.2 [11]. Proteins identified as “reverse”, “only identified by site”, or “potential contaminants” hits were removed. The LFQ intensities were log2-transformed and z-scored before performing a hierarchical clustering of the proteins with the following settings: Euclidean distance, complete linkage, based on a preprocessing with k-means with 300 clusters, 10 iterations, and 1 restart. Under- and overexpressed proteins were selected based on z-scores being exclusively ≤ − 1 or ≥ + 1, respectively. The gene IDs corresponding to the under- and overexpressed proteins were then imported into the PANTHER v.13.1 gene ontology classification system [12].

## Results and discussion

The aim of this study is to create a pipeline where MSI data is used to accurately guide the LMD system for further analysis on the very same tissue section with potential applications in biomedical research. This pipeline consists in several steps which are listed in ESM, Protocol S1.

### MSI of breast cancer tissue

MSI of lipids was performed on a fresh-frozen breast cancer tissue section, which was mounted onto an LMD-compatible membrane slide to be able to use the same section for MSI and LMD. We used a high-pressure MALDI mass spectrometer, which leaves most of the membrane unaffected and therefore usable by an LMD system. Three co-registration steps had to be performed to couple the obtained MSI data to the LMD system (Fig. 1).

### Co-registration of MSI and LMD

The first step consisted of co-registering the MSI data to the high-resolution optical image of the tissue with matrix (Fig. 1(I)). To be most accurate and overcome limitations in vendor-shipped software, this was achieved by directly matching the coordinates of three manually selected MSI pixels with their corresponding visible laser-shot landmarks in the optical image (ESM, Fig. S1a). An affine geometric transformation was derived from these points and used to transform all MSI pixels into the coordinate system of the optical image (and vice versa). The co-registration error was estimated by counting the number of pixels in the optical image (~ 2 μm) from the center of the laser shot landmark to the corresponding MSI pixel (ESM, Fig. S1b). The error was on average 7.9 μm in *x* and 4 μm in *y* (Table 1).

**Table 1:** Co-registration errors

Co-registration of …	… optical image—MSI data	… optical image—H&E image	… optical image—LMD (magnification × 5)	Maximum error (assuming additive effects)
Error in *x* (μm)	7.89 ± 4.06 SD	1.39 ± 0.33 SD	3.46 ± 2.62 SD	12.74 ± 4.84 SD
Error in *y* (μm)	3.96 ± 4.32 SD	1.39 ± 0.50 SD	7.39 ± 3.78 SD	12.74 ± 5.76 SD

*MSI*, mass spectrometry imaging; *LMD*, laser microdissection; *H&E*, hematoxylin and eosin; *SD*, standard deviation

The second co-registration needed is between the optical and the H&E images in order to incorporate the histological information into the data analysis (Fig. 1(II)). The manual selection of three control point pairs was based on the same features of the fiducial markers in both optical images, which enabled the creation of an affine geometric transformation. The manual selection of co-registration points induces an error which was estimated by performing five manual co-registrations of the optical image with itself and calculating the average of the Euclidean distances between all original and transformed positions. The average error was 1.4 μm for both *x* and *y* (Table 1)*.*

The fiducial markers were also used to translate coordinates from the optical image to the coordinate system of the LMD (Fig. 1(III)). Three landmark points were manually selected in the optical image for this purpose. One of these is used as origin point, which means that all other coordinates belonging to the two remaining landmarks and the regions of interest (ROIs) are recalculated with respect to that reference point. These new coordinates were then written into an XML file to import the ROIs into the LMD software. During the import process, the exact same teaching points, previously defined in the optical image, had to be selected in the LMD live image of the slide. The error of this co-registration step was estimated by comparing expected and observed distances between cut co-registered shapes and micrometer-sized Tipp-Ex spots (~ 3 μm) in the LMD live image (ESM, Fig. S2). The average error was 3.5 μm in *x* and 7.4 μm in *y* (Table 1). The error was evaluated at × 5 magnification which corresponds to the lowest magnification level of the LMD. While this magnification enables a large field of view, which was necessary for the co-registration, it also provides the lowest detail level for matching the previously selected landmarks in the optical image to their representations in the LMD live image.

It is important to mention that in all co-registration steps, the teaching points were selected manually which influence the alignment quality of both co-registered objects. Moreover, the estimation of the reported errors in the optical images was based on visual evaluation which also introduces bias and error. We addressed this issue by repeating each co-registration at least three times (Table 1).

In summary, the co-registration error for each step was below 10 μm. We note that these errors could be reduced by using a higher magnification in the LMD (ESM, Table S1) or a higher resolution of the optical images (ESM, Table S2). We also want to point out that the reported errors are control point based and do not account for additional errors due to morphological deformations during histological staining. We expect that powerful elastic image registration using the tissue’s own features, which must therefore be clearly visible in highly resolved MSI images, could decrease these detrimental effects [13]*.* Although it is not possible to evaluate if the errors in the three steps are additive or subtractive, we expect the co-registration error to be below 13 μm in both dimensions, considering additive error components (Table 1).

### Detection of molecular distinct tumor populations by MSI

In this study, the usefulness and feasibility of the established MSI-LMD link are demonstrated by the investigation of intratumor heterogeneity in a breast cancer sample.

**Fig. 2:**
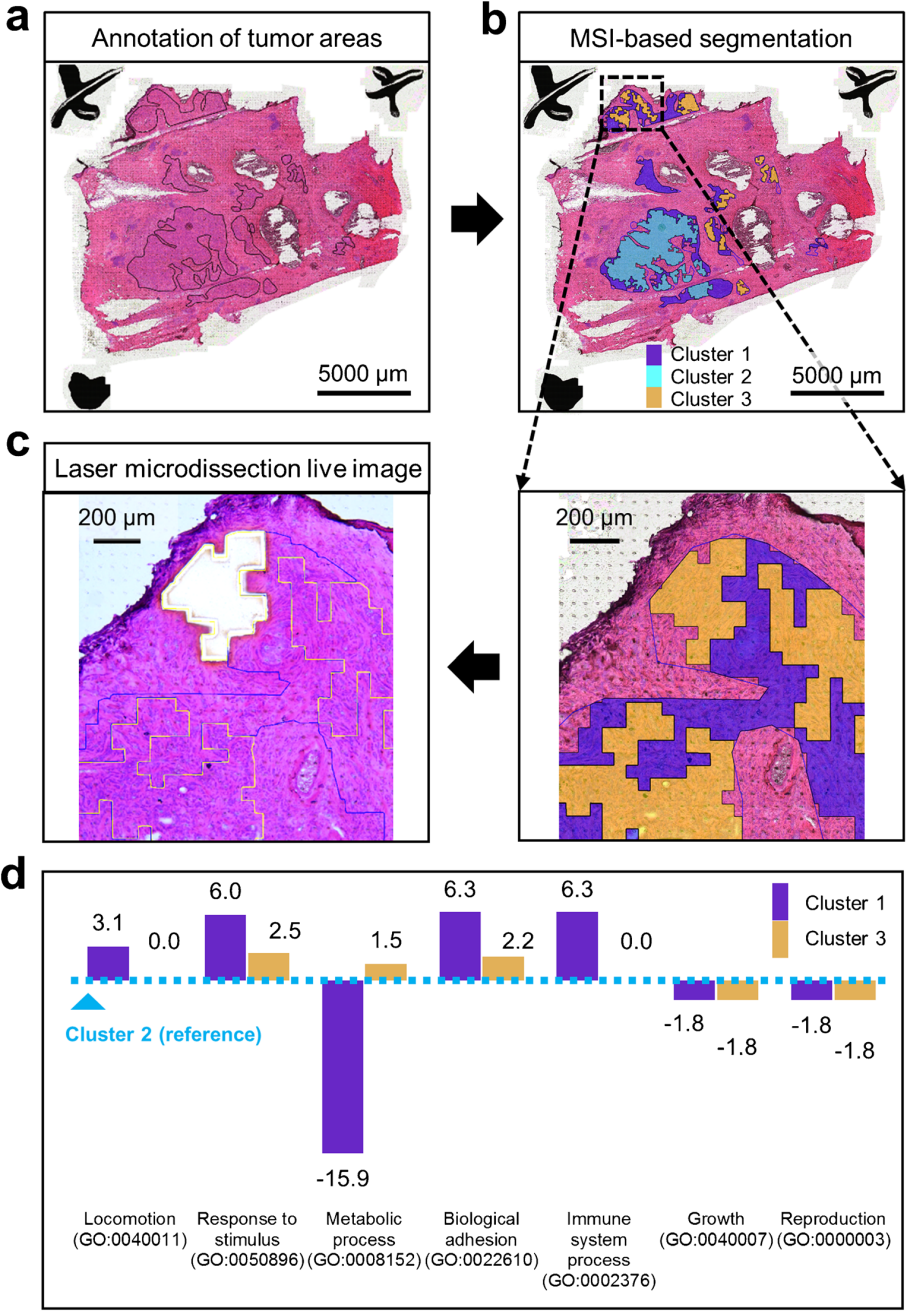
Laser microdissection of MSI-defined intratumor segments subsequently characterized by microproteomics. (a) First, tumor areas were annotated by a pathologist on the H&E image. (b) Then, mass spectrometry imaging (MSI) lipid data of the tumor was used to spatially segment the tumor areas into three clusters using non-negative matrix factorization. After image processing of the segments including smoothing and boundary detection (ESM, Figs. S3 and S4), the segmentation image was upscaled to the resolution of the H&E image. (c) The segments’ boundaries were finally transferred to the LMD software using the previously established co-registration pipeline. Each MSI cluster was then microdissected by the LMD system for the subsequent microproteomics analysis. This resulted in the identification and label-free quantification of over *1000 common proteins.* Cluster exclusive over- and under-expressed proteins were submitted to gene ontology analysis (ESM, Fig. S5). (d) shows selected differences in molecular functions between the clusters (in percentage points with respect to cluster 2)

In order to investigate intratumor heterogeneity, a pathologist first delineated the tumor areas in the H&E-stained tissue section (Fig. 2(a)). For high accuracy, these annotations were done on the high-resolution image in the scan viewer software. The annotations and the H&E image were then imported into MATLAB and co-registered to the MSI data. This allows exclusive selection of tumor-specific MSI data.

As previously demonstrated, unsupervised multivariate analysis can be used to reveal intratumor heterogeneity through MSI-based tissue segmentation [2]. Here, the tumor areas were partitioned applying the NNMF algorithm to the MSI data. The average Silhouette coefficient was maximized in order to determine the optimal number of segments. In a range from two to five, the optimal number of segments was found to be three (ESM, Table S3).

Image processing was performed on the segmentation results in order to increase the practicability of microdissection. This included at first a smoothing of the segmentation image using the *imopen* MATLAB function with a 2 × 2 square as structuring element (ESM, Fig. S3). The segmentation image was then divided into three binary images, each depicting the pixels belonging to one of the segments, and which were further processed individually (ESM, Fig. S4). This included the removal of impurities within the binary image by deleting small areas (*≤* 30 pixels in the 4-connected neighborhood) using *bwareaopen* and filling holes in the 8-connected neighborhood using *imfill* (ESM, Fig. S4). The individual binary images were then warped to the dimensions of the histological image using *imwarp* with nearest-pixel interpolation (Fig. 2(b)). Once upscaled, the last step of the image processing was to detect the external boundaries of all regions belonging to a segment using *bwboundaries* (ESM, Fig. S4). Finally, the boundary coordinates of each MSI segment were recalculated with respect to the selected origin point present in the optical image and were used to generate the XML file in the LMD-compatible format.

### Laser microdissection and microproteomics characterization of MSI segments

The ROIs were available and visible in the field of view of the LMD software after import of the MSI-based ROI information into the LMD system (Fig. 2(c)). These ROIs were then microdissected (Fig. 2(c)) and collected into tubes for a further molecular characterization of the different tumor cell populations represented by the MSI segments using quantitative 2D-LC-MS/MS microproteomics*.*

A total of 1426 common proteins were identified among the MSI segments (ESM, Table S4). After LFQ normalization, log2-transformation, and standardization, 1040 proteins remained to characterize the molecular properties of the different tumor subpopulations (ESM, Table S5). As expected when analyzing samples from the same tissue section, few proteins (on average 25.3) were found exclusively up- or downregulated in each MSI segment when using a z-score threshold of + 1 and − 1, respectively (ESM, Fig. S5a). The subsequent functional annotation based on those proteins showed that the three MSI segments mainly differed from each other in processes related to metabolic processes, biological adhesion, immune system process, response to stimulus, and locomotion (Fig. 2(d) and ESM, Fig. S5b). The observation of functional differences between the different tumor regions confirms the presence of molecular and functional intratumor heterogeneity in this breast cancer sample.

## Conclusion

Mass spectrometry imaging (MSI) continues its development toward a higher spatial resolution and cellular sensitivity but still lacks chromatographic separation to mitigate ion suppression. A transfer of spatial MSI information to a laser microdissection (LMD) system for the targeted isolation of cellular material followed by bottom-up proteomics must be correspondingly accurate. As MSI systems nowadays achieve resolutions around 10 μm, co-registration errors should ideally not be larger than a few MSI pixels. Here we achieved an accurate coupling of MSI to LMD with co-registration errors on a single MSI pixel level, namely below 13 μm. While here we focused on microproteomics experiments, the excised samples could be analyzed by any other omics technology compatible with MSI. Our approach ultimately brings together spatial and molecular information for a better understanding of in situ molecular mechanisms in complex tissues.

## Electronic supplementary material

ESM 1 (PDF 2.01 MB)

ESM 2 (XLSX 247 kb)

ESM 3 (XLSX 157 kb)
