# Supplementary material for: Precise co-registration of mass spectrometry imaging, histology, and laser microdissection-based omics
Source: Anal Bioanal Chem. 2019 Jul 1;411(22):5647–53. doi: 10.1007/s00216-019-01983-z (PMC6704276; doi:10.1007/s00216-019-01983-z)
Supplement: Supplementary file 1 — (PDF 2.01 MB) [file 216_2019_1983_MOESM1_ESM.pdf]

Analytical and Bioanalytical Chemistry

Electronic Supplementary Material

Precise co-registration of mass spectrometry imaging, histology, and laser microdissection-based omics

Frédéric Dewez, Marta Martin-Lorenzo, Michael Herfs, Dominique Baiwir,
abriel Mazzucchelli, Edwin de Pauw, Ron M.A. Heeren, Benjamin Balluff

Additional files available under 10.1007/s00216-019-01983-z.

Protocol S1. Step-by-step description of the co-registration between mass spectrometry imaging, histology, and the laser microdissection system.

1. Mass spectrometry imaging (MSI) experiment
    1.1. Tissue sectioning
    1.2. Slide preparation
        1.2.1. Matrix application
        1.2.2. Fiducial markers with Tipp-Ex
    1.3. MSI data acquisition
    1.4. High-resolution optical scan of the slide with matrix
    1.5. Washing off matrix
    1.6. Hematoxylin and eosin (H&E) staining
    1.7. High-resolution optical scan of H&E-stained slide
2. MATLAB processing
    2.1. Import of MSI data, high-resolution optical and H&E image
    2.2. Co-registration of MSI data with optical image via laser shot landmarks
    2.3. Co-registration of optical image with H&E image via fiducial markers
    2.4. Annotation of H&E image
    2.5. Segmentation on tumor-related spectra only with non-negative matrix factorization (NNMF)
    2.6. Image processing of NNMF segmentation results
        2.6.1. Smoothing
        2.6.2. For every binary MSI segmentation image separately do
            2.6.2.1. Remove small objects
            2.6.2.2. Fill holes
            2.6.2.3. Upscale to resolution of H&E image
            2.6.2.4. Detect boundaries
    2.7. Recalculation of coordinate boundaries via fiducial markers
    2.8. Generation of an XML file as an input for laser microdissection (LMD) system
3. LMD system
    3.1. Import of XML file generated by 2.8
    3.2. Co-registration to LMD system via the exact same fiducial markers as 2.3
    3.3. Cutting out of regions-of-interest

Table S1 Co-registration error of the optical image with the laser microdissection system depending its magnification

Co-registration of …	… Optical image – LMD (magnification 10x)	… Optical image – LMD (magnification 20x)	… Optical image – LMD (magnification 40x)
**Error in X (µm)**	5.91 ± 6.90 SD	3.48 ± 4.26 SD	3.88 ± 5.09 SD
**Error in Y (µm)**	4.25 ± 5.61 SD	3.57 ± 3.81 SD	2.64 ± 3.25 SD

Abbreviations used: LMD, laser microdissection; SD, standard deviation

Table S2 Co-registration error of optical image with mass spectrometry imaging

Co-registration of …	… Optical image – MSI data (downscaled 1:8)	… Optical image – MSI data (downscaled 1:4)
**Error in X (µm)**	9.97 ± 5.63 SD	7.89 ± 4.06 SD
**Error in Y (µm)**	6.25 ± 2.64 SD	3.96 ± 4.32 SD

Abbreviations used: MSI, mass spectrometry imaging; SD, standard deviation

Table S3 Optimal number of NNMF clusters (k) evaluated by the average Silhouette coefficient

Number of NNMF clusters	k=2	k=3	k=4	k=5
**Average Silhouette coefficient**	0.243	0.293	0.277	0.099

Tables S4 and S5 see separate Excel files:

Table S4 Groups of proteins identified using MaxQuant software. The proteins were identified and quantified using the label-free quantification (LFQ). These following settings were applied: Uniprot reviewed human database, trypsin digestion with two maximum missed cleavage sites, methionine oxidation as variable modification and carbamidomethyl cysteine as fixed modification, a minimal peptide length of seven amino acids, at least two peptides per protein (of which at least one is unique), and a maximum false discovery rate of 1%. The label-free intensities were normalized using the MaxLFQ algorithm.

Table S5 Groups of proteins used for data analysis in the Perseus software. Proteins identified as ‘reverse’, ‘only identified by site’ or ‘potential contaminants’ hits were removed. After LFQ normalization in MaxQuant, intensities were log2-transformed and standardized. This left 1040 common proteins to characterize the molecular properties of the different tumor subpopulations.

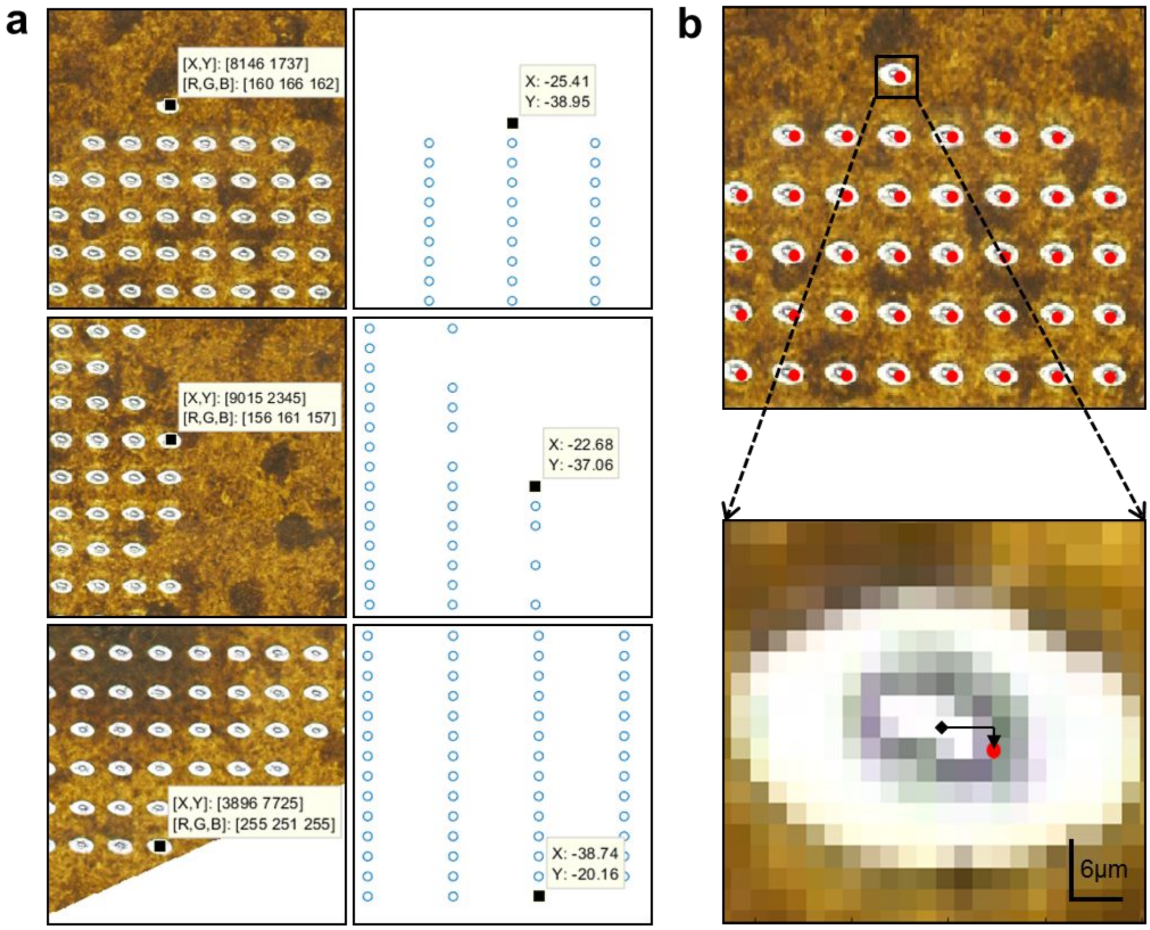

Fig. S1 Co-registration of MSI to the high-resolution optical image. (a) The co-registration between the MSI data and the optical image was done via an affine geometric transformation between three landmarks of the laser shots in the matrix, that are visible in the high-resolution optical image (a, left column), and the corresponding MSI pixels (a, right column). (b) The estimation of the co-registration error was assessed by counting the number of pixels (pixel size ≈ 2 μm) for x and y in the optical image separating the center of the laser shot landmark from the corresponding MSI pixel plotted on top of the optical image

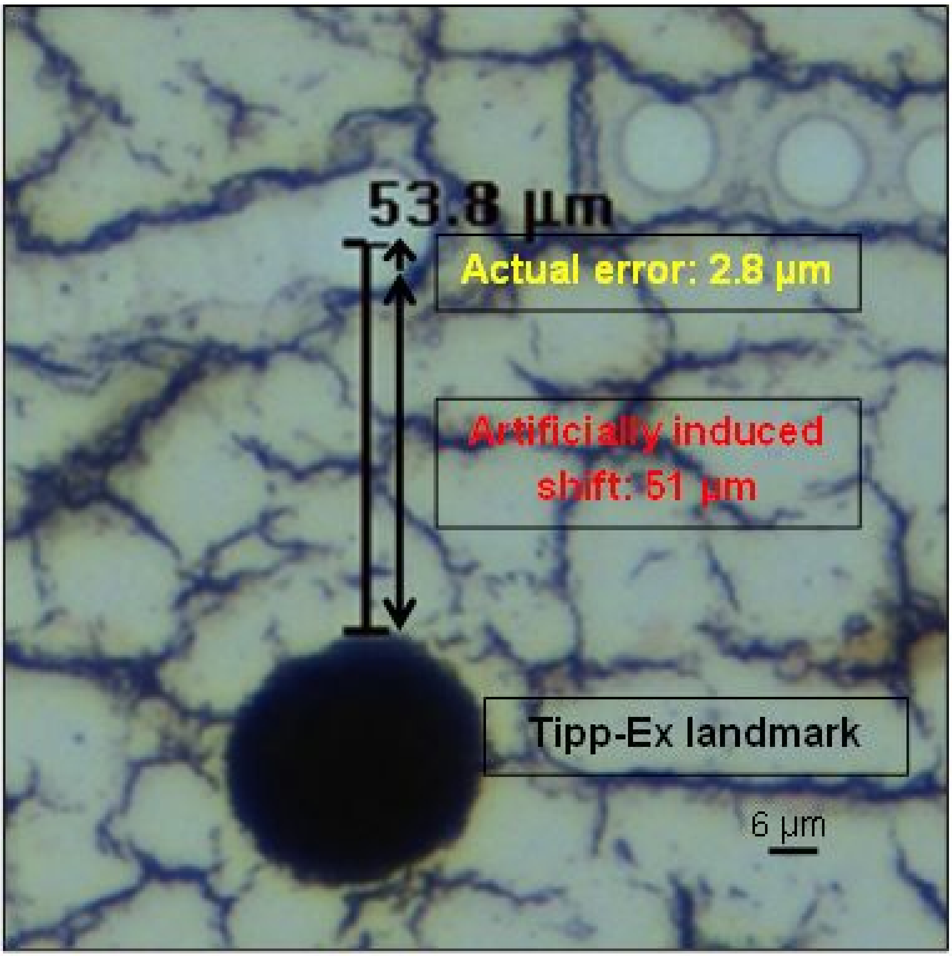

Fig. S2 Estimation of the LMD co-registration error at 5x magnification. The co-registration error between optical image and the LMD was determined by creating virtual shapes in the optical image with a known distance to nearby Tipp-Ex spots. These shapes were then cut by the laser of the LMD at 5x magnification. The co-registration error was then evaluated at 10x magnification by measuring the distance between the Tipp-Ex spot and the laser landmark in the tissue of the cut shapes

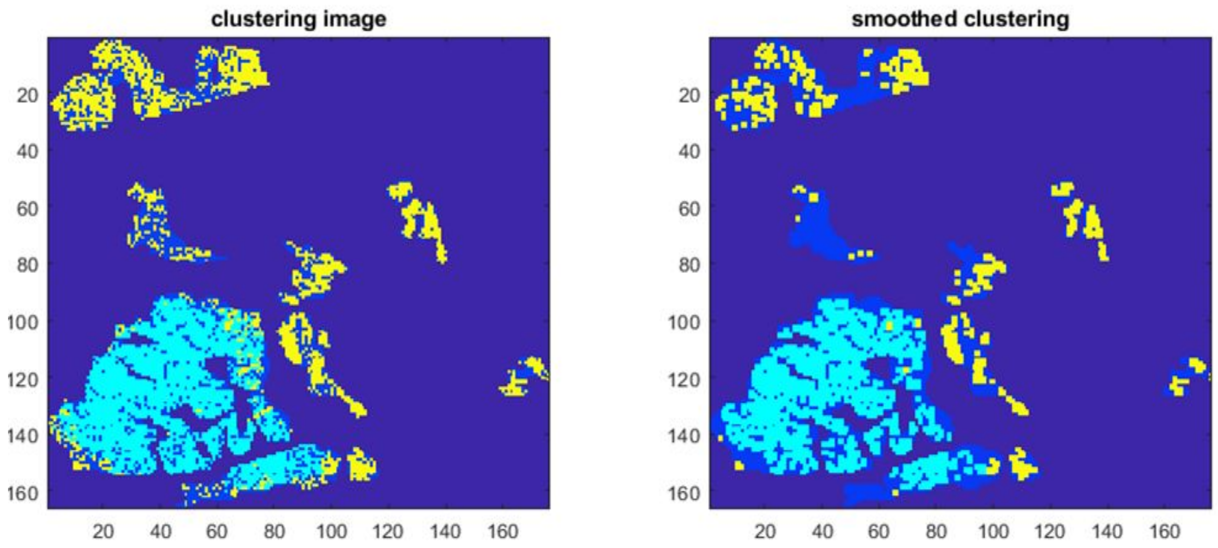

Fig. S3 Smoothing of the NNMF segmentation image. Image processing was performed on the raw segmentation results (left image) to increase the viability of microdissection. This included at first a smoothing of the segmentation image using the *imopen* Matlab function with a 2x2 square as structuring element. The result is shown in the right image

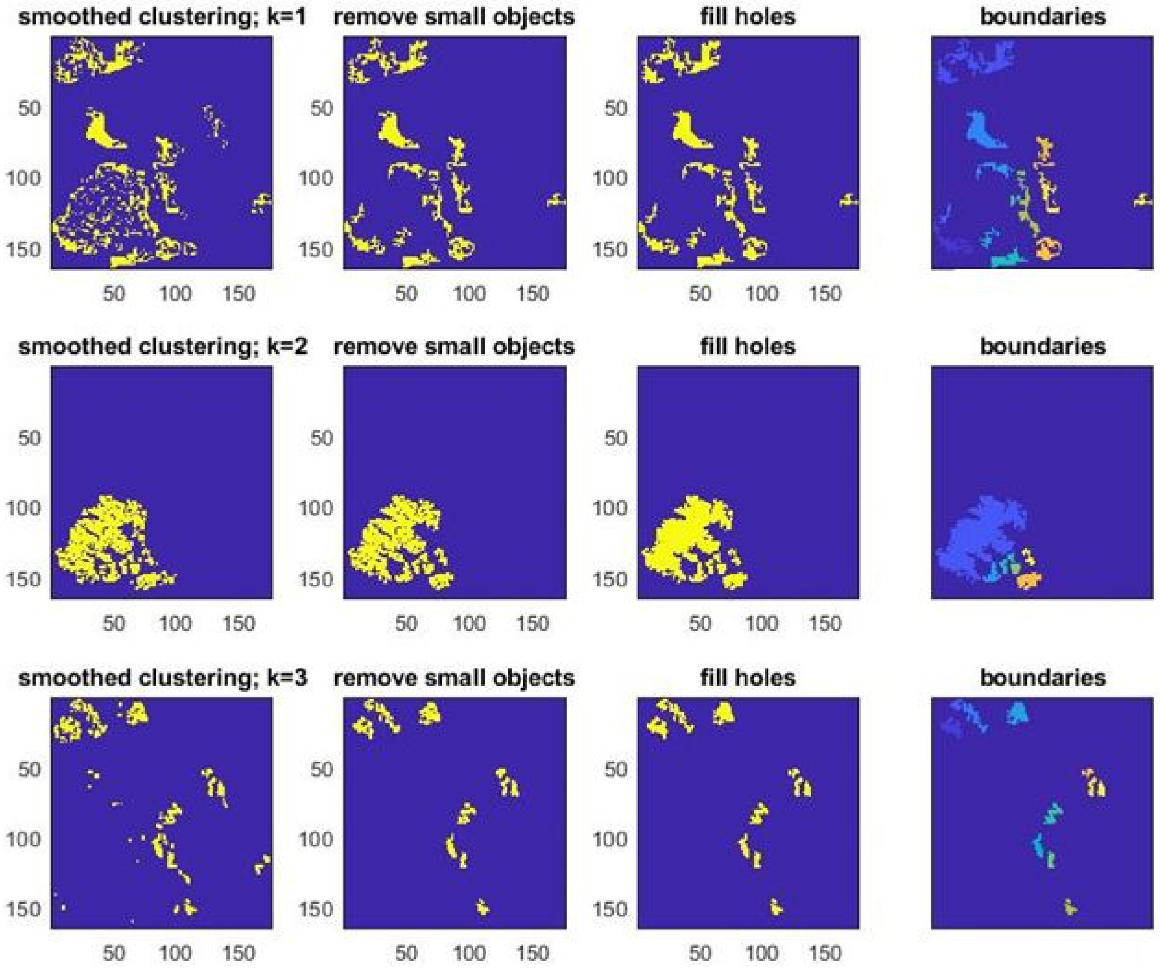

Fig. S4 Image processing after NNMF segmentation. Several steps of image processing on the smoothed segmentation image were further needed in order to detect the boundaries of each clusters (left to right columns). First, the smoothed segmentation image was divided into three binary images, each depicting the pixels belonging to one of the clusters (leftmost column). Each of these was further processed individually. Impurities were removed by deleting small areas (≤30 pixels in the 4-connected neighborhood) using *bwareaopen* (second column) and by filling holes in the 8-connected neighborhood using *imfill* (third column). The individual binary images were then warped to the dimensions of the histological image using *imwarp* with nearest-pixel interpolation. Once up-scaled, the last step of the image processing was to detect the external boundaries of all segments belonging to each cluster using *bwboundaries* (rightmost column)

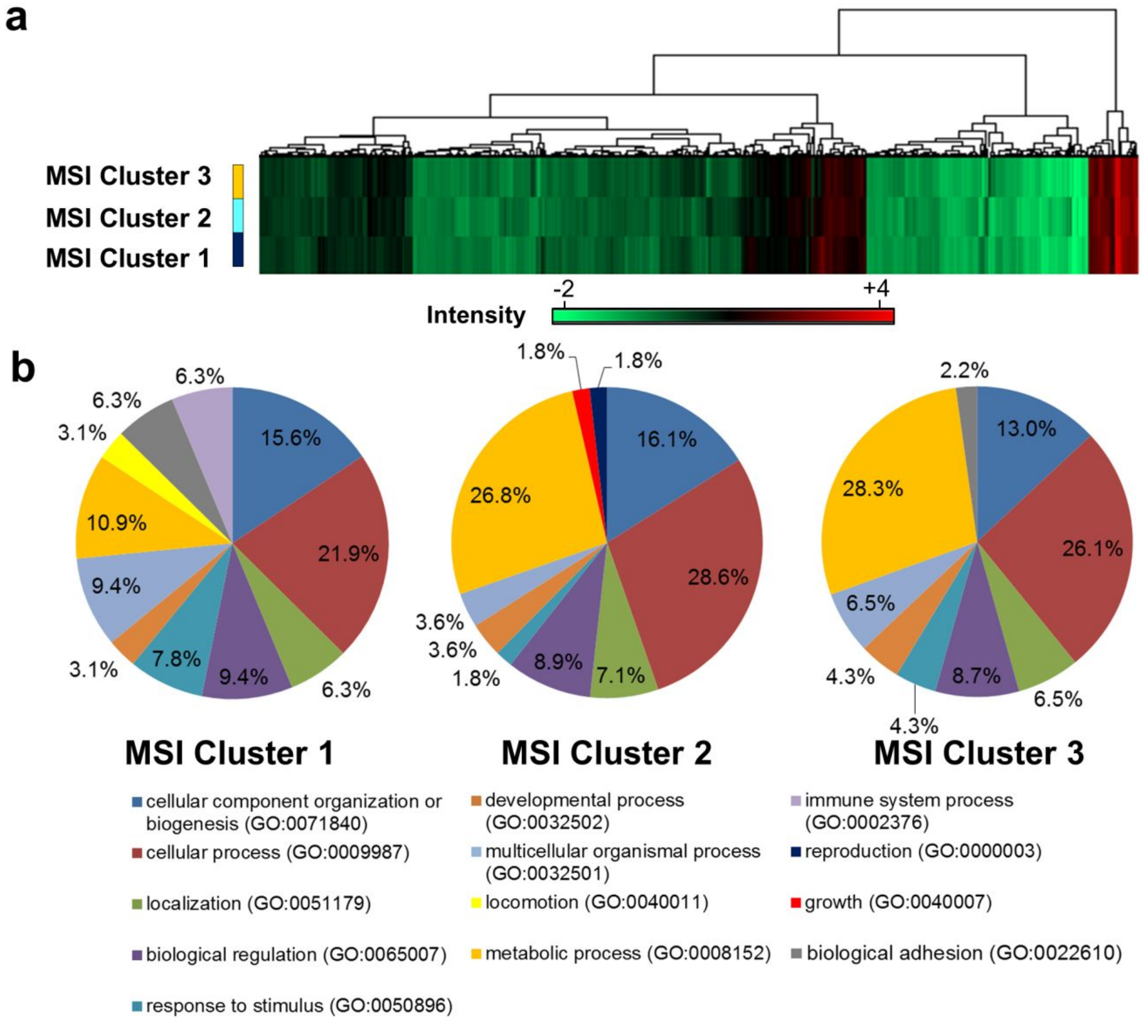

Fig. S5 Microproteomics characterization of MSI-defined intra-tumoral clusters. LMD was performed for each of the MSI-defined intra-tumoral clusters by cutting out 0.3mm^2-equivalent material for each MSI-cluster for microproteomics analysis. This resulted in the identification and label-free quantification of over *1400 common proteins.* (a) Hierarchical clustering was performed on the log2-tranformed and z-scored data (*1040 proteins remained*) to group MSI-clusters and proteins by expressing similarity. (b) Cluster exclusive over- and under-expressed proteins (z-scores ≥+1 and ≤-1, respectively) were submitted to gene ontology analysis to determine differences in predominant molecular function between the three MSI clusters, and therefore between the different breast cancer subpopulations
